# Polymorphism of Keratin Gene *KRT71* and Its Relationship with Wool Properties in Gansu Alpine Fine-Wool Sheep

**DOI:** 10.3390/ani15142028

**Published:** 2025-07-10

**Authors:** Fangfang Zhao, Zhaohua He, Hongxian Sun, Jiqing Wang, Xiu Liu, Zhiyun Hao, Mingna Li, Shaobin Li

**Affiliations:** 1 Gansu Key Laboratory of Herbivorous Animal Biotechnology, Faculty of Animal Science and Technology, International Wool Research Institute, Gansu Agricultural University, Lanzhou 730070, China; hezh1250148718@163.com (Z.H.); sunhx@st.gsau.edu.cn (H.S.); wangjq@gsau.edu.cn (J.W.); liux@gsau.edu.cn (X.L.); haozy@gsau.edu.cn (Z.H.); limn@gsau.edu.cn (M.L.); lisb@gsau.edu.cn (S.L.); 2 College of Biology and Food, Shangqiu Normal University, Shangqiu 476000, China

**Keywords:** *KRT71*, wool characteristics, hair follicle development, Gansu alpine fine-wool sheep

## Abstract

Keratin plays a crucial role in wool formation. Conducting polymorphism studies on key keratin genes is helpful in identifying key SNP sites that might influence wool traits. In this research, kompetitive allele-specific PCR (KASP) genotyping and protein immunofluorescence techniques were used to explore the polymorphisms of the *KRT71* gene in Gansu alpine fine-wool sheep, analyze the relationship between the gene polymorphisms and wool production traits, and examine the expression and localization of the KRT71 protein in the hair follicles of fine-wool sheep. The results indicated that there were two single-nucleotide polymorphisms (SNPs) in the 5′ UTR and exon 9 of the *KRT71* gene, named SNP1 (C.-7G/C) and SNP2 (C.1500G/A), respectively. Regarding SNP1, the mean stable length (MSL) of *GG* genotype individuals was significantly longer than that of *GC* genotype individuals (*p* < 0.05). Similarly, for SNP2, the MSL of *GG* genotype individuals was significantly greater than that of *GA* genotype individuals (*p* < 0.05). Moreover, the KRT71 protein showed moderate positive expression in the cuticle, outer root sheath, and sebaceous gland. It had strong positive expression in the inner root sheath, while no positive expression was detected in the hair medulla and hair papilla. In summary, the sheep *KRT71* gene could be an important candidate gene for improvements in wool length.

## Simple Summary

As the structural proteins of wool, the keratin family’s structural and functional changes are likely to cause changes in wool traits. Previous studies have found that KRT71 is a differentially expressed protein in different types of wool, but there is still a lack of research on its expression localization and gene variation. This study analyzed the expression localization and gene polymorphism of *KRT71* using protein immunofluorescence and kompetitive allele-specific PCR methods. The results showed that KRT71 had strong positive expression in the inner root sheath, and the variation in *KRT71* showed a notable association with the wool length.

## 1. Introduction

Wool, a raw textile material with a long-standing history, is well-liked by consumers due to its softness, warmth, flame retardancy, elasticity, and wearing comfort [1]. In the consumer market, wool textiles and other wool-based products, like wool sound insulators in cosmetics, wool antimicrobial bandages, and lanolin in medical supplies, have become indispensable in daily life [2,3]. The quality and yield of wool serve as crucial factors in enhancing the economic efficiency of the wool industry [4,5]. Relevant research has indicated that wool quality traits (such as fineness, crimp, length, etc.) have moderate–high heritability, which implies that variations in wool traits might be significantly affected by genetic factors [6,7]. Hence, when the nutritional levels and environmental factors remain constant, genetic factors are significant determinants of wool quality. At the same time, protein-coding genes play vital regulatory roles in the development of various traits in both plants and animals [8].

Keratin and keratin-associated protein genes play crucial roles in hair formation in both humans and animals [9,10,11]. Research has revealed that keratins not only offer structural support to cells but also participate in intracellular signaling pathways. These pathways are responsible for regulating metabolic processes and promoting the growth of epithelial cells [12]. The *KRT71* gene, which is part of the keratin family, belongs to the type II keratins. In humans, it is located on chromosome 12, while, in sheep, it is situated on chromosome 3. Moreover, the *KRT71* gene is primarily expressed in the root sheath of the hair follicle. Mutations in this gene might serve as the genetic foundation for curly hair and oligohemorrhagia disease [13]. It has been discovered that base mutations in the *KRT71* gene could interact with the counterparts of this gene in type I keratin. This interaction subsequently results in curly hair in animals or humans [14]. Additionally, Duan et al. demonstrated that the mRNA expression levels of genes like *KRT1*, *KRT6A*, and *KRT71* in the skin of curly-haired Yanshan cashmere goats were significantly higher compared to those of non-curly-haired goats. Furthermore, cashmere goats with curly-fiber cashmere could produce cashmere with a finer diameter [15]. The aforementioned studies imply that the *KRT71* gene may have a significant impact on hair formation and hair follicle development. Nevertheless, the influence of mutations in the gene base sequence of the *KRT71* gene on hair follicle development and wool traits in fine-haired goats requires further exploration.

Gansu alpine fine-wool sheep hold significant economic value and ecological status in the alpine pastoral areas of the Qinghai–Tibet Plateau and Qilian Mountains of China. Nevertheless, when compared with fine-wool sheep breeds like Australian Merino and Chinese Merino, there remains a disparity in the wool quality of Gansu alpine fine-wool sheep [16]. Traits including the fineness of wool and the number of hair follicles all have an impact on the wool price, and these traits are co-regulated by multiple genes [17]. Consequently, it is essential to thoroughly explore the functional genes associated with the regulation of wool traits in Gansu alpine fine-wool sheep from a genetic perspective and subsequently uncover the gene functions.

In this research, based on our pre-sequencing results [18], we further selected the candidate gene *KRT71* for gene polymorphism detection. We employed kompetitive allele-specific PCR (KASP) genotyping technology and protein immunofluorescence to examine the polymorphisms of the *KRT71* gene in Gansu alpine fine-wool sheep and the relationship between gene polymorphisms and wool production traits. Additionally, we investigated the expression localization of the KRT71 protein in the hair follicles of fine-wool sheep. This study enriches the molecular genetic data on economic traits in fine-wool sheep.

## 2. Materials and Methods

### 2.1. Ethics Statement

The animals used in the study were all from a farm in Tianzhu Zangzu Autonomous County, China. Informed consent was obtained from the animal owners. The animal experiments were approved by the Gansu Agricultural University Ethics Committee under the code GSAU-ETH-AST-2021-028.

### 2.2. Experimental Animals and Sample Collection

In this research, blood and wool samples were obtained from 315 one-year-old Gansu alpine fine-wool sheep ewes in Songshan Township, Tianzhu Tibetan Autonomous County to identify single-nucleotide polymorphisms (SNPs) in the *KRT71* gene. All these sheep were kept under the same nutritional levels and feeding environment. The collected blood samples were stored at −20 °C for the subsequent extraction of blood DNA. Wool samples were taken from the left scapula area of the sheep to measure various wool economic traits. Additionally, a one-year-old Gansu alpine fine-wool sheep ewe was chosen, and a skin sample was collected from its left scapula. This skin sample was then fixed in a 4% paraformaldehyde solution at room temperature for protein immunofluorescence identification. When the samples were collected, the wool fibers were in the growth stage of the hair follicle.

### 2.3. Determination of Wool Production Traits

Wool samples were used for the determination of various wool economic traits, such as the mean fiber diameter (MFD), coefficient of variation of fiber diameter (CVFD), fiber diameter standard deviation (FDSD), mean fiber curvature (MFC), comfort factor (CF), MSL, and mean stable strength (MSS), etc. The wool analysis was carried out with reference to the National Standard of the People’s Republic of China for Sheep Wool (GB1523-2013) [19].

### 2.4. Protein Immunofluorescence Localization Analysis

Sheep skin tissue was immersed in 4% paraformaldehyde for fixation over a period of 48 h. Subsequently, an immunofluorescence assay was carried out to detect the KRT71 protein, following the method described by Diao et al. [20]. For the immunofluorescence detection of the KRT71 protein, the primary antibody employed was K71 rabbit anti-rabbit (Product Item No.: GTX107343, GeneTex, Irvine, CA, USA), and the secondary antibody was FITC-labeled goat anti-rabbit (Product Item No.: GB22303, Servicebio, Wuhan, China). The microscopic images were observed and analyzed using the CaseViewer 2.4 (3DHISTECH, Budapest, Hungary) image analysis software. Positive expression scores were assigned to the cuticle, dermal papilla, sebaceous gland, inner root sheath, outer root sheath, and hair medulla sites within three fields of view of the sections.

### 2.5. Blood Genomic DNA Extraction

Whole blood samples from Gansu alpine fine-wool sheep were thawed and genomic DNA was extracted according to the steps of the EasyPure^®^ Blood Genomic DNA Kit (Beijing All Style Gold Biotechnology Co., Ltd., Beijing, China). Agarose gel electrophoresis was employed to examine the quality of the DNA, while the NanoDropTM Lite Ultra-Micro Spectrophotometer (Thermo Fisher Scientific Inc., Waltham, MA, USA) was utilized to measure the concentration and purity of the genomic DNA. The extracted genomic DNA was stored at −20 °C for preservation.

### 2.6. Detection of SNPs in the KRT71 Gene

The ENSEMBL database was employed to forecast the polymorphisms of the *KRT71* gene. Based on the prediction of SNPs in this gene, the target fragments of the *KRT71* gene to be amplified were chosen. The gene sequence of sheep *KRT71* (accession number: NC_056056.1) was retrieved from the NCBI database. Subsequently, specific primers were designed using the Premier 5.0 software (Version 5.0, Premier Biosoft International, Palo Alto, CA, USA), and the details of these primers are presented in Table 1. These primers were synthesized by Sangon Biotech in Shanghai, China.

In this research, genomic DNA from 20 Gansu alpine fine-wool sheep was selected. The target fragments of the *KRT71* gene were individually amplified. Agarose gel electrophoresis was utilized to assess the integrity of the amplified products. After this, the amplified products were sent to Sangon Biotech in Shanghai, China for one-generation Sanger sequencing. Once the sequencing was completed, the SNPs of the target fragments were screened by integrating the sequencing peak maps with the base sequence information.

### 2.7. Genotypic Detection of SNP Sites

The number of SNP sites within the target fragment of the *KRT71* gene was determined. Subsequently, the genomic DNA of all Gansu alpine fine-wool sheep, along with the details of the identified SNP sites, was submitted to Gentides Biotechnology Co., Ltd. (Gentides, Wuhan, China) for KASP genotyping. A microplate reader equipped with a fluorescence resonance energy transfer (FRET) probe was employed to gather fluorescence data. Online software (http://www.snpway.com/snpdecoder/, accessed on 7 July 2025) was utilized to generate genotyping maps.

### 2.8. Statistical Analysis

Microsoft Excel 2021 was employed for the collation and statistical analysis of the SNP genotype data. Based on the method reported by Botstein et al. [21], the allele frequencies (AF), genotype frequencies (GF), chi-squared values (**χ**2), gene heterozygosity (He), homozygosity (Ho), polymorphism information content (PIC), and effective number of alleles (Ne) were calculated.

The detected SNPs were subjected to the chi-squared (**χ**2) test to assess their Hardy–Weinberg equilibrium status. The online software SHEsis (http://shesisplus.bio-x.cn/SHEsis.html, accessed on 8 July 2025) was utilized to analyze the linkage disequilibrium (LD) at the gene SNP site.

A general linear model (Yijk = µ + Gi + Ɛijk) in SPSS 26.0 (version 26.0, IBM Corp., Armonk, NY, USA) was used to analyze the relationships between genotypes and wool production traits. Y represents the phenotypic value of the trait; µ is the overall mean; Gi is the genotype effect; and Ɛijk is the random error. The results are presented as the mean ± standard error, and a *p*-value < 0.05 indicates significant differences.

## 3. Results

### 3.1. Localization of KRT71 Protein in Sheep Skin Tissue

**Figure 1:**
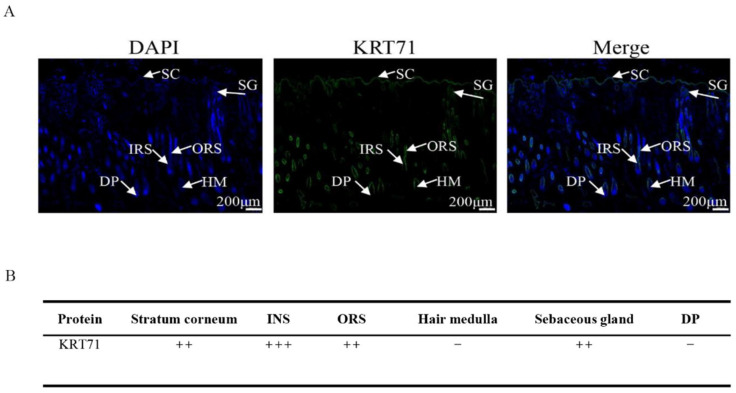
Localization of KRT71 in the skin tissue of Gansu alpine fine-wool sheep. (**A**) KRT71 protein immunofluorescence; (**B**) positive intensity of KRT71 protein in skin tissue. KRT71 fluorescence staining is shown in green, while DAPI-labeled cell nucleus fluorescence staining is shown in blue. DP: dermal papilla; SC: stratum corneum; SG: sebaceous gland; IRS: inner root sheath; ORS: outer root sheath; HM: hair medulla. –: no positive expression; ++: moderate positive expression; +++: strong positive expression.

The immunofluorescence results showed that there were some differences in the intensity of KRT71 positivity in different parts of the skin and hair follicles of Gansu alpine fine-wool sheep during the wool growth period. Among them, the KRT71 protein was moderately positively expressed at the cuticle, outer root sheath, and sebaceous gland of the skin; strongly positively expressed in the inner root sheath; and not positively expressed in the hair medulla and dermal papilla (Figure 1).

### 3.2. Results of SNP Site Detection of KRT71 Gene in Gansu Alpine Fine-Wool Sheep

**Figure 2:**
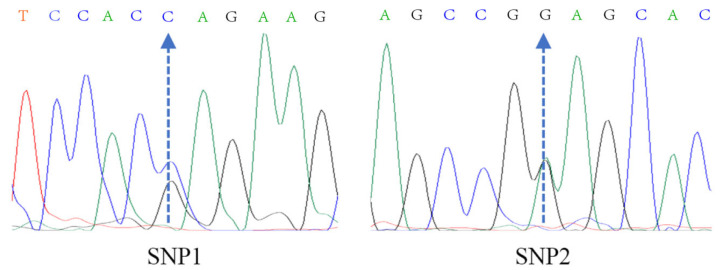
Sequencing peak map of SNP screening for *KRT71* gene in Gansu alpine fine-wool sheep.

The results showed that there were two SNPs in the two target fragments of the *KRT71* gene in Gansu alpine fine-wool sheep (Figure 2). Among them, the SNPs in the 5′ UTR region and exon 9 region of the *KRT71* gene were named SNP1 (C.-7G/C) and SNP2 (C.1500G/A), respectively (Table 2). The SNP at exon 9 was a synonymous mutation.

### 3.3. Genotyping Results of SNPs in KRT71 Gene

**Figure 3:**
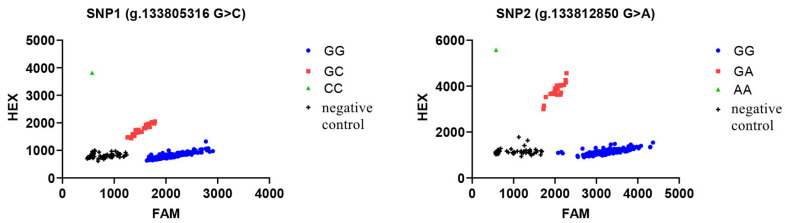
Genotyping results of *KRT71* gene in Gansu alpine fine-wool sheep. The green and blue groups clustered near the horizontal and vertical axes represent pure heterozygotes, respectively. The red groups clustered separately between the two are heterozygotes, and the black groups clustered near the intersection of the axes are negative controls.

KASP technology was used to type the SNPs of the *KRT71* gene in Gansu alpine fine-wool sheep. As shown in Figure 3, the two SNPs were both polymorphic and showed three genotypes. Meanwhile, the gene and genotype frequency statistics showed that the dominant genotypes of SNP1 and SNP2 were GG (92.36%) and GG (91.95%), and the dominant alleles were G (0.960) and G (0.958), respectively (Table 3).

### 3.4. Population Genetic Polymorphism Analysis of the KRT71 Gene

The population genetic polymorphism results showed that the two SNPs in the *KRT71* gene had high purity (Ho > 0.5), and the SNP with the highest number of effective alleles was SNP2 (Ne = 1.087). In addition, the SNP loci all showed low polymorphism (PIC < 0.25). The heterozygosity (He) results showed that the heterozygote frequencies at the SNP1 and SNP2 loci were 0.076 and 0.080, respectively (Table 4).

### 3.5. Association Analysis of KRT71 Genotype with Wool Production Traits

As shown in Table 5, the two SNPs of the *KRT71* gene all showed a significant association with the MSL (*p* < 0.05). Specifically, in SNP1, the MSL of GG genotype individuals were significantly longer than that of GC genotype individuals (*p* < 0.05). Similarly, in SNP2, the MSL of GG genotype individuals was significantly longer compared to that of GA genotype individuals (*p* < 0.05).

### 3.6. Linkage Disequilibrium Analysis of SNP Loci in the KRT71 Gene

**Figure 4:**
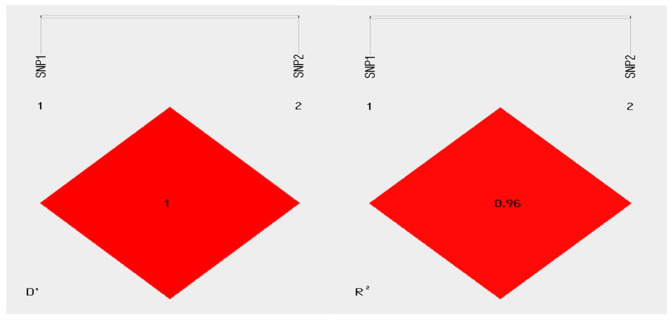
Linkage disequilibrium analysis of SNPs in *KRT71* gene of Gansu alpine fine-wool sheep. D’ = 0, R^2^ = 0, indicates that the loci of the two SNPs are in a state of complete linkage equilibrium; D’ = 1, R^2^ = 1, indicates that the loci of the two SNPs are in a state of complete linkage disequilibrium.

In this study, two indicators (D*’* and R^2^ values) were combined to determine the linkage disequilibrium between the SNP loci of the *KRT71* gene. The results showed that there was strong linkage disequilibrium between the two SNP loci of the *KRT71* gene (D’ = 1, R^2^ = 0.96) (Figure 4).

## 4. Discussion

Detecting SNPs of functional genes is an effective approach to identifying the key SNP sites of genes. This method has been extensively utilized in analyzing the polymorphisms of functional genes associated with various animal production traits, including wool, meat, milk, and fat deposition. Chai et al. discovered that a base mutation (c.-364A/G) in the promoter fragment of the *KRT34* gene might influence the wool economic traits of Merino sheep, such as the mean fiber diameter (MFD), fiber diameter standard deviation (FDSD), and mean staple length (MSL) [22]. Zhao et al. demonstrated that five SNP loci (g. 105914953 G > A, g. 105922232 T > C, g. 105922244 A > G, g. 1059223 A > T, and g. 105922340 G > T) in the FGF5 gene were significantly correlated with traits like the wool length, wool weight, and growth performance in fine-wool sheep [23]. Base mutations (c.600 G/A and c.652 + 93 C/A) in the exon 3–4 region of the *KRT85* gene were related to wool production in Merino sheep [24]. There was a strong correlation between polymorphisms (G > A) in the FASN gene and the wool fiber diameter [25]. Moreover, mutations in SLIT3 and ZNF280B were found to be correlated with the wool fiber diameter of alpine merino sheep [26]. In summary, the research method of detecting gene SNPs and conducting an association analysis between these SNPs and various animal production traits is feasible. It is more beneficial for application in production practice, thereby facilitating improvements in various production traits of farm animals.

The efficient and convenient screening of candidate genes is of great significance for subsequent gene validation. At present, there are numerous methods for the screening of functional genes, including whole-genome resequencing, transcriptome analysis, proteome analysis, DNA methylation (WGBS), RNA methylation (such as m6G, m5C, m7G, etc.), and other epigenetic modifications, along with other emerging technological tools [27]. In this research, after integrating the pre-sequencing results and referring to the relevant literature [18,28], we carried out further screening and selected the *KRT71* gene for in-depth investigation. We explored the potential relationships between these functional genes and wool economic traits through gene and protein expression localization and polymorphism detection.

Genes and proteins exhibit tissue-specific expression patterns, which subsequently lead to diverse biological functions [29,30]. Immunofluorescence, a potent tool in biological research and medical diagnosis, boasts high sensitivity in antigen detection. It can precisely localize and detect specific proteins or antigens and has been extensively applied in various biological and medical research fields [31,32]. Moreover, immunofluorescence techniques enable the direct visualization of target antigens within cells or tissue, offering intuitive visual data that aid in comprehending antigen distribution and localization. Consequently, to explore the expression and localization of KRT71 in skin hair follicles, this study was carried out. The results indicated that the KRT71 protein showed strong positive expression in the inner root sheaths of skin hair follicles. Relevant research has demonstrated that the length and curvature of the inner root sheath significantly influence the shape of wool [33]. This finding further implies that the *KRT71* gene might play a crucial role in hair follicle development as well as wool growth. Therefore, it is essential to conduct research on its genetic polymorphisms and explore the relationship between SNPs of the *KRT71* gene and wool economic traits.

SNPs could induce changes in wool traits through multiple molecular and biological mechanisms. These primarily encompass the following: SNPs within coding regions can modify the structure and function of proteins, influence gene expression levels, and cause chain imbalances and are related to quantitative trait loci (QTL); key gene variants can impact signaling pathways; there are alterations in epigenetic regulation (such as SNPs in methylation-sensitive loci and variants in histone modification loci), as well as indirect physiological effects [34,35]. Moreover, apart from being present in coding regions, SNPs are highly distributed in non-coding regions. They can also be detected in non-coding RNAs, introns, or the 5′ and 3′ untranslated regions (UTRs) [36]. Even though these changes do not lead to the production of modified proteins associated with specific diseases, they can still affect RNA–protein interactions by altering the secondary structure of RNA [37,38]. Although the change in the *KRT71* gene SNP identified in this study is a synonymous mutation and does not result in any amino acid substitutions, it may affect protein expression or structure. It has been reported that synonymous mutations may affect the rate of mRNA translation, thereby potentially altering the way in which proteins fold [39]. The observed effect of the *KRT71* gene may also be due to its linkage to other KRTs on the same chromosome. KRT71 belongs to the keratin family and its biological significance needs to be further investigated.

In the polymorphism analysis of genes, conducting an association analysis between genotypes and production traits is beneficial in identifying crucial loci of SNPs, which might play a role in enhancing the economic traits of farm animals [40]. In this research, we discovered that the variation in the *KRT71* gene was related to the MSL. The MSL is one of most important wool economic traits and is closely linked to the wool processing performance. Wool with a long MSL is more commercially desirable as it tends to be easier to spin, leads to fewer stoppages, and ultimately can form stronger and more even yarns when compared to shorter wool [41]. This suggests that, if the wool length is used as the improvement target, individuals with the GG genotype will be more popular in the wool industry.

Even though the association between variations in the *KRT71* gene and wool curvature did not show a significant difference, in both SNP1 and SNP2, there was still a trend in which the wool curvature of GC genotype and GA genotype individuals was higher than that of GG genotype individuals. Moreover, numerous previous studies have also demonstrated a strong correlation between *KRT71* gene variants and the curliness of animal hair [13,42,43]. In subsequent studies, researchers could consider increasing the sample size to further examine the associations between this gene variation and wool production traits.

## 5. Conclusions

This research indicates that variations in the *KRT71* gene are related to the mean stable length of wool in Gansu alpine fine-wool sheep, while the KRT71 protein is strongly positively expressed mainly at the root sheath within the hair follicle in fine-wooled sheep.

## Figures and Tables

**Table 1:** Primer information of *KRT71* gene.

Gene	Position	Forward Primer (5′ → 3′)	Reverse Primer (5′ → 3′)	Amplification Product Length (bp)
*KRT71*	5′ UTR	TTCATGTTGTTTCAGAGGTGGGGAG	CTCAGGGAAGACTATACGGGAAACCTT	835
	E9	TCTCTGCCATATTGTCCCCAGTGG	TCAGGAAGACACAGTCTGGGAGAAGTG	535

**Table 2:** SNP-related information for *KRT71* gene.

Gene	SNP	Position	Physical Position/bp	Variation Type	Amino Acid Change
*KRT71*	SNP1	5′ UTR	g.133805316	G → C	No change
	SNP2	Exon 9	g.133812850	G → A	No change

**Table 3:** Genotype frequency and allele frequency of *KRT71* gene.

Genotype	Genotype Frequency(n)			Allele Frequency		χ^2^	*p*-Value
SNP1	*GG*	*GC*	*CC*	G	C	0.617	0.432
	0.924 (278)	0.073 (22)	0.003 (1)	0.960	0.040		
SNP2	*GG*	*GA*	*AA*	G	A	0.470	0.493
	0.920 (274)	0.077 (23)	0.003 (1)	0.958	0.042		

**Table 4:** Analysis of genetic parameters of *KRT71* gene population.

SNP	Ho	He	PIC	Ne
SNP1	0.923	0.076	0.074	1.083
SNP2	0.919	0.080	0.077	1.087

**Table 5:** The associations between gene variations in *KRT71* and wool traits.

SNP	Genotype	MFD (μm)	FDSD	CVFD (%)	MSL (mm)	MFC (Number/mm)	CF (%)	MSS (cN/dt)
SNP1	*GG*	21.508 ± 0.156	5.624 ± 0.073	25.142 ± 0.213	80.047 ± 0.768 ^a^	104.998 ± 0.815	87.482 ± 0.805	13.982 ± 0.303
	*GC*	21.726 ± 0.628	5.395 ± 0.204	24.766 ± 0.551	75.454 ± 1.841 ^b^	106.138 ± 2.989	89.285 ± 3.034	15.848 ± 1.051
SNP2	*GG*	21.516 ± 0.158	5.619 ± 0.074	25.139 ± 0.214	79.963 ± 0.768 ^a^	104.913 ± 0.818	87.566 ± 0.805	13.986 ± 0.305
	*GA*	21.733 ± 0.601	5.368 ± 0.196	24.645 ± 0.539	75.304 ± 1.766 ^b^	106.386 ± 2.861	89.500 ± 2.900	15.806 ± 1.005

Note: The shoulder labels of data in the same column with different lowercase letters indicate significant differences (*p* < 0.05).

## Data Availability

The authors affirm that all data necessary to confirm the conclusions of the article are present within the article, figures, and tables.
